# *PyExtal*: a Python package for quantitative convergent-beam electron diffraction

**DOI:** 10.1107/S1600576726001469

**Published:** 2026-03-31

**Authors:** Hsu-Chih Ni, Robert Busch, Jian-Min Zuo

**Affiliations:** a Department of Materials Science and Engineering, University of Illinois Urbana–Champaign, Urbana, IL 61801, USA; b Materials Research Laboratory, University of Illinois Urbana–Champaign, Urbana, IL 61801, USA; c Monash Centre for Electron Microscopy, Monash University, Clayton, VIC3800, Australia; d Department of Materials Science and Engineering, Monash University, Clayton, VIC3800, Australia; DESY, Hamburg, Germany

**Keywords:** quantitative convergent-beam electron diffraction, large-angle CBED, LACBED, structure factors, refinement algorithms, electron diffraction, multiple scattering

## Abstract

Quantitative convergent-beam electron diffraction (QCBED) enables the measurement of structure factors and other structural parameters with high accuracy using dynamical diffraction theory. To do this, rigorous diffraction simulations and iterative intensity refinement are required. We have developed *PyExtal*, a Python-based software package for QCBED. *PyExtal* supports refinement of both small- and large-unit-cell crystals through automated computation workflows and flexible data handling of convergent-beam electron diffraction (CBED) and large-angle CBED patterns. The performance of *PyExtal* is demonstrated by using silicon and yttrium iron garnet as benchmark systems, with refined silicon structure factors showing excellent agreement with previous experimental results. As a computational platform, *PyExtal* provides a direct basis for experimental mapping of electron charge density in crystals and facilitates broader applications of dynamical-diffraction-based quantitative crystallographic analysis.

## Abstract 2

We introduce a Python package with parallel computing capabilities designed for the refinement of convergent-beam electron diffraction (CBED) or large-angle CBED patterns.

## 1. Introduction

The principle of quantitative convergent-beam electron diffraction (QCBED) is to extract precise crystallographic information from the complex intensity distributions recorded in experimental convergent-beam electron diffraction (CBED) patterns. Unlike selected-area electron diffraction with discrete diffraction spots, CBED produces diffraction discs whose internal intensity varies sensitively with changes in incident beam direction due to multiple or dynamical scattering of electrons within the crystal (Spence & Zuo, 1992 ▸). By quantitatively matching experimental and simulated intensity distributions, it is possible to determine (1) crystal structure factors, (2) atomic positions and lattice parameters, (3) specimen thickness, (4) Debye–Waller factors, and (5) strain fields (Zuo *et al.*, 1999 ▸; Tsuda & Tanaka, 1999 ▸; Zuo *et al.*, 1998 ▸; Saunders *et al.*, 1999 ▸; Nakashima *et al.*, 2011 ▸; Wu *et al.*, 2020 ▸; Lin *et al.*, 2025 ▸).

The ability of QCBED to determine structure factors with high accuracy has attracted considerable interest for the study of electron charge density and bonding effects. However, most QCBED investigations to date have focused on crystals with relatively small unit cells, such as Al, Cu_2_O, CdS_2_ and TiO_2_ (Tsuda & Tanaka, 1999 ▸; Zuo *et al.*, 1999 ▸; Jiang *et al.*, 2003 ▸; Nakashima *et al.*, 2011 ▸). This fact arises primarily from two practical limitations. First, the beam convergence angle must remain smaller than the Bragg angle to prevent disc overlap in CBED, a condition that is more easily satisfied for small-unit-cell crystals. Second, the focused electron beam leads to an increase in the electron fluence and thus limits the application of CBED to beam-sensitive materials. Additionally, as the unit-cell size increases, the number of diffracted beams grows rapidly, leading to a substantial increase in computational time.

More recent developments in three-dimensional electron diffraction (3DED) (Kolb *et al.*, 2007 ▸; Zhang *et al.*, 2010 ▸; Jones *et al.*, 2018 ▸; Gemmi *et al.*, 2019 ▸) have made a tremendous impact on the determination of structural parameters such as atomic coordinates. Dynamical refinement of 3DED data improves the precision of structure determination (Palatinus *et al.*, 2015*a* ▸; Palatinus *et al.*, 2015*b* ▸; Klar *et al.*, 2023 ▸). However, whether these methods can extract accurate structure factors like CBED is still an open question. There have been attempts to refine partial charges using 3DED with promising results (Mahmoudi *et al.*, 2025 ▸; Dominiak *et al.*, 2025 ▸).

Compared with 3DED, the large-angle rocking-beam electron diffraction (LARBED) method has the potential to improve the quality of electron diffraction datasets with high angular resolution and overcome the limitations of conventional CBED (Koch, 2011 ▸; Beanland *et al.*, 2013 ▸; Busch *et al.*, 2024 ▸). In LARBED, an electron beam with a small convergence angle is systematically tilted, or ‘rocked’, over a wide angular range, while a diffraction pattern is recorded at each tilt (Busch *et al.*, 2024 ▸). The resulting four-dimensional dataset maps the diffracted intensities as functions of both scattering angle and the beam incidence direction at a high sampling rate with step sizes of less than 0.2 mrad. From this dataset, large-area CBED (LACBED) patterns can be reconstructed for individual reflections, providing an extended view of the diffraction space for both small- and large-unit-cell crystals.

Quantitative matching of CBED patterns demands precise knowledge of the diffraction geometry, specimen thickness and electron optical potential and is best achieved through iterative refinement that incorporates many-beam dynamical diffraction effects (Spence & Zuo, 1992 ▸). Several refinement algorithms have been developed for this purpose (Zuo & Spence, 1991 ▸; Saunders *et al.*, 1995 ▸; Nüchter *et al.*, 1998 ▸; Birkeland *et al.*, 1998 ▸; Zuo, 1999 ▸; Ogata *et al.*, 2004 ▸). Among them, the *Extal* algorithm was designed for quantitative analysis of off-zone-axis CBED patterns (Zuo, 1999 ▸), whereas the *MBFIT* algorithm is optimized for zone-axis or near-zone-axis CBED patterns (Tsuda & Tanaka, 1999 ▸). However, these existing structured computer programs are limited in computational flexibility and scalability, motivating the development of new refinement frameworks capable of handling the large, complex datasets produced by modern LARBED experiments.

Here, we introduce *PyExtal*, a Python-based software package developed for the refinement of structure factors and crystallographic parameters from electron diffraction patterns. The refinement strategy implemented in *PyExtal* is based on the *Extal* algorithm originally implemented in Fortran by Zuo (1998 ▸). *PyExtal* extends this refinement strategy with three major objectives: (1) to modernize the *Extal* algorithm for contemporary computing environments, enabling broader accessibility and programming flexibility; (2) to expand its applicability to LACBED patterns and thus to datasets acquired using LARBED; and (3) to exploit parallel computing capabilities available in modern multi-core systems. By achieving these goals, *PyExtal* aims to provide a comprehensive and extensible platform for accurate, quantitative structure factor determination based on electron diffraction.

## 2. Principles of *PyExtal*

### 2.1. Least-squares fitting

*PyExtal* performs quantitative refinement to extract precise structural information from experimental CBED patterns. Rather than analyzing individual diffraction peak intensities, the program simulates CBED patterns based on a diffraction model and iteratively refines the model parameters to achieve optimal agreement with the experimental data. The initial diffraction model is constructed using an approximate scattering potential, typically derived from independent atoms placed at the crystallographic sites. Additional model parameters include the crystal orientation, thickness, incident beam wavevector and detector characteristics. Multiple scattering of electrons is fully accounted for through dynamical diffraction theory, which is used to compute the simulated intensity distributions. The inclusion of dynamical diffraction effects enhances the analysis sensitivity and improves the CBED measurement accuracy.

The refinement proceeds by least-squares optimization of model parameters to minimize or maximize a goodness-of-fit (GOF) function, which includes the standard 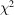 function, the *R* factor often used in crystallography and the cross-correlation function used for image matching. The 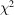 function is the weighted sum of squared intensity differences between the measured and simulated patterns, as defined by 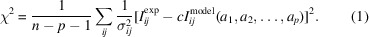
Here, 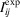 is the experimental intensity recorded in the CBED pattern and 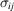 is the estimated standard deviation, *i* and *j* are the pixel coordinates on the detector, *n* is the total number of data points, and *a* and *p* are the adjusted parameter and the number of parameters. 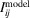 is the calculated diffraction intensity with the model parameters 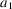 to 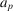, and *c* is the normalization coefficient. The optimum 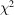 has a value close to unity, which is obtained when the differences between theory and experiment are normally distributed and σ is correctly estimated.

### 2.2. Experimental CBED patterns and their preparation

The quality of experimental CBED patterns is critical to the success of QCBED refinement. In general, inelastic scattering background must be removed from diffraction patterns, although a differential technique developed by Nakashima & Muddle (2010 ▸) can mitigate this requirement. Key considerations in acquiring CBED patterns for quantitative analysis include the choice of diffraction condition (*e.g.* zone-axis versus off-zone-axis), minimization of geometric distortions arising from projection lens aberrations and the energy filter, control of detector characteristics such as the point spread function (PSF) and noise, and optimization of specimen thickness. Preparation of CBED data for refinement further requires accurate calibration of the accelerating voltage, determination of the diffraction camera length and correction for the detector PSF through deconvolution. Detailed discussions of these experimental and data-processing procedures can be found in the literature (Zuo, 1998 ▸; Zuo, 1999 ▸; Friis *et al.*, 2003 ▸).

The LACBED patterns analyzed here were acquired using the LARBED method described by Busch *et al.* (2024 ▸). In this approach, a series of point-like diffraction patterns are recorded using a nearly parallel beam in a double-rocking configuration originally developed for precession electron diffraction. Systematic beam rocking over a two-dimensional grid is controlled by the on-board detector control unit of a direct electron detector (Busch *et al.*, 2024 ▸), while the microscope is operated in a transmission electron microscope (TEM) dark-field tilt mode. At each grid point, a diffraction pattern corresponding to a single beam tilt is recorded, producing a four-dimensional dataset in the coordinate space of (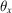, 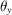, 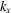, 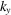), where 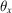 and 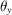 represent the beam tilt angles and 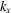 and 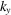 the reciprocal-space detector coordinates. Diffraction intensities are extracted by integrating the Bragg peaks and subtracting the diffuse background, yielding LACBED patterns for individual reflections. A notable advantage of the LARBED method is its ability to effectively separate diffuse scattering from Bragg diffraction, and therefore it can be used for quantitative analysis without the need of energy filtering.

The standard deviation of the experimental intensity can be estimated using the measured detector quantum efficiency (DQE) in the case of energy-filtered CBED by using (Zuo, 2000 ▸)
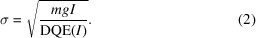
Here, *I* is the measured diffraction intensity, *m* is the mixing factor obtained from the measured modulation transfer function (MTF) and *g* is the gain of the detector. For LARBED, the diffraction intensity is obtained from the point-like diffraction pattern using the peak integration and background subtraction method described above. The variance of the intensity can be estimated using
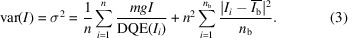
Here, the first term represents the variance for the integrated intensity, in line with the method used for CBED. The second term is the variance contribution of the background subtraction, which is taken as a standard deviation of background intensity values. *m* for the EMPAD detector used in this work can be measured following the method described by Zuo (2000 ▸), while DQE can be taken as 0.94 for a 300 kV accelerating voltage (Tate *et al.*, 2016 ▸). 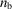 is the number of pixels in the background intensity estimation and 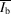 is the mean background intensity.

### 2.3. Calculation of theoretical intensity

The Bloch wave method is used to calculate theoretical diffraction intensities. For crystals, this approach is preferred because of its accuracy and flexibility, whereas the multi-slice method or related techniques are better suited for the simulation of interfaces, defects or other structural inhomogeneities. A detailed description of the Bloch wave formalism is given by Spence & Zuo (1992 ▸). Within this formalism, when CBED discs do not overlap, the diffraction intensity for the incident beam characterized by 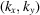, corresponding to the reflection 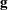, is given by 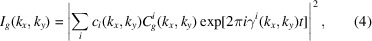
where the excitation coefficients 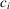 are determined by matching the incident wave and waves inside the crystal of thickness *t* at the entrance surface. The eigenvalue of the *i*th Bloch wave 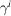 and eigenvector component 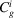 are obtained by solving
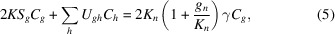
where 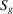 is the excitation error at the incident beam direction of 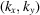 for the reflection 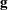, 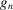 and 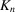 are the components of 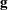 and incident wavevector 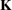 along the surface normal direction 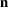, and 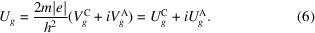

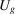
 is the electron structure factor, which is related to the Fourier coefficient of the scattering potential (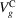), including the contribution of the absorption potential 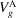. Here, *m* and *e* are the mass and charge of the electron, respectively, and *h* is Planck’s constant. The electron structure factor can be approximately calculated using the following formula:
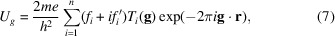
using the known atomic scattering factor *f* and absorption factor 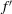 and the ‘temperature factor’ *T*, with 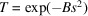 for isotropic displacement parameters [where *B* is the Debye–Waller factor and 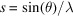]. The solution of equation (5) generally converges as the number of beams included in the diagonalization increases. In *PyExtal*, the strong beams are included in the diagonalization, while weak beams are treated by perturbation using the Bethe potential method (Spence & Zuo, 1992 ▸). In this implementation, an initial list of beams is selected using the criteria of maximum **g** length, maximum excitation error and their perturbational strength, and then additional criteria are used for selecting strong beams (Zuo & Weickenmeier, 1995 ▸).

To model diffraction intensities, both the detector response and the background intensity must be considered. A general expression including both factors is 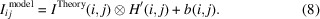
Here, the theoretical intensity, which is calculated for each detector pixel (*i*, *j*), is convoluted with the detector response function *H*′ plus the background intensity *b*. For a CBED pattern after the removal of the detector response function using deconvolution, *H*′ can be approximated by the delta function. For LARBED, *H*′ can be interpreted as the profile of the incident beam in reciprocal space. The background intensity *b*, in general, is slowly varying. *PyExtal* implements several background intensity models, including a constant background for each reflection.

## 3. Implementation

The *PyExtal* software package features a flexible Python-based interface that communicates with a Bloch wave simulation engine implemented in Fortran for numerical optimization and analysis. This hybrid architecture combines the strengths of both languages: the computational efficiency of Fortran is utilized for the intensive tasks involved in Bloch wave calculations, particularly matrix diagonalization, while Python provides an interactive and user-friendly scripting environment that streamlines control, automation and refinement workflows.

### 3.1. Package architecture

**Figure 1:**
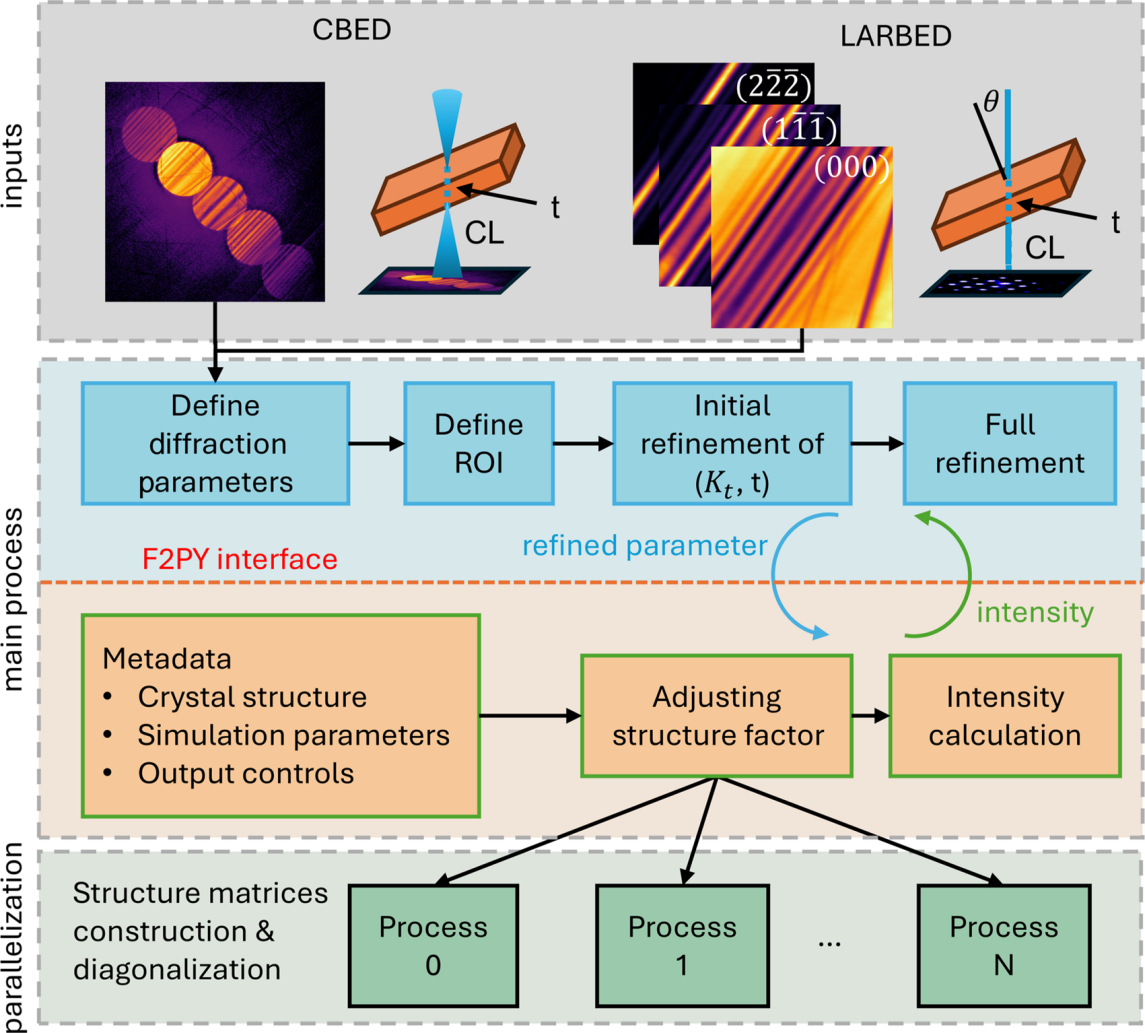
Architecture of *PyExtal*. Diffraction intensity and geometry are taken as parameters for the refinement. The blue boxes show the workflow in the Python interface and orange boxes show how the underlying Bloch engine works. The construction and diagonalization of structure matrices with different incident beam directions are parallelized.

The architecture of *PyExtal* is illustrated in Fig. 1 ▸. The CBED or LACBED patterns are used as the experimental input and refined in the workflow depicted by the blue boxes in the figure. The refinement process begins with the definition of the experimental parameters, which includes the diffraction geometry of the experimental diffraction pattern(s) and the (*hkl*) indices for LACBED patterns. This is followed by the selection of a region of interest (ROI) for refinement. First, a correlation-based refinement is performed, followed by the intensity-based refinement. These refinement steps are iterative loops that repeatedly call the Bloch simulation engine with new model parameters to progressively improve the fit to the experimental patterns.

The computationally intensive Bloch wave simulation is parallelized using the message passing interface (MPI), which spawns a group of sub-processes, and the Bloch wave simulation for different incident beam directions is then distributed among these sub-processes. This design provides efficient parallelization of the Bloch wave calculations while maintaining a simple single-process user interface in Python, making it well suited for interactive environments like *Jupyter Notebooks* (https://jupyter.org/).

The Bloch wave simulation code, adapted from that reported by Spence & Zuo (1992 ▸), uses *LAPACK* (Anderson *et al.*, 1999 ▸) for matrix diagonalization. The Fortran source code is compiled into a shared library using *f2py* (Peterson, 2009 ▸), which creates a seamless interface with Python. Through this interface, variables in the Bloch wave simulation can be accessed and manipulated directly from Python, enabling an effective integration of high-level scripting and high-performance computation.

### 3.2. Bloch simulation configuration file

All parameters, *i.e.* metadata, for Bloch wave simulation, including definition of the crystal structure, experimental conditions and calculation settings, are specified in a text-based .dat configuration file as shown in Fig. 1 ▸. This file uses a sequential keyword-based format. The file is organized into several mandatory and optional sections.

(1) *Crystal structure definition*. This data section defines the properties of the crystal. It includes the unit-cell parameters (cell), space group (spg), atomic positions (atom) in fractional coordinates, Debye–Waller factors and site occupancy.

(2) *Experimental and diffraction parameters*. This section includes following parameters: the accelerating voltage (hv), the crystal orientation relative to the beam (zone, norm), the incident beam direction (kt) and the electron-beam convergence angle (conv).

(3) *Beam selection*. This section defines which reflections are included in the Bloch wave simulation. This can be done either by manually listing all beams (hkl) or by using an automatic selection method (sele) based on geometric criteria such as maximum excitation error (sgmax) and reciprocal lattice vector length (gmax). The criteria are described by Zuo & Weickenmeier (1995 ▸).

(4) *Calculation and output control*. These parameters control the weak-beam selection and output of the Bloch wave simulation. They include the thresholds for the selection of weak beams (sgmin, omeg). Weak beams are included in the Bloch wave simulation using the Bethe potential perturbation method. For output control, either a simulated objective aperture (aper) or a manual list (out) is used.

(5) *Structure factor adjustment (optional*). Specific structure factors can be manually adjusted from their default independent atom model (IAM) values (Doyle & Turner, 1968 ▸) using the adj keyword.

### 3.3. Coordinate systems and region of interest definition

Mapping the simulated diffraction pattern onto the experimental one is a critical step in the refinement process. In *PyExtal*, this is achieved by establishing the transformation between the experimental sampling (detector) coordinates and the reciprocal space used in simulation. To facilitate this transformation, we introduce an intermediate coordinate system termed the theoretical sampling coordinates.

**Figure 2:**
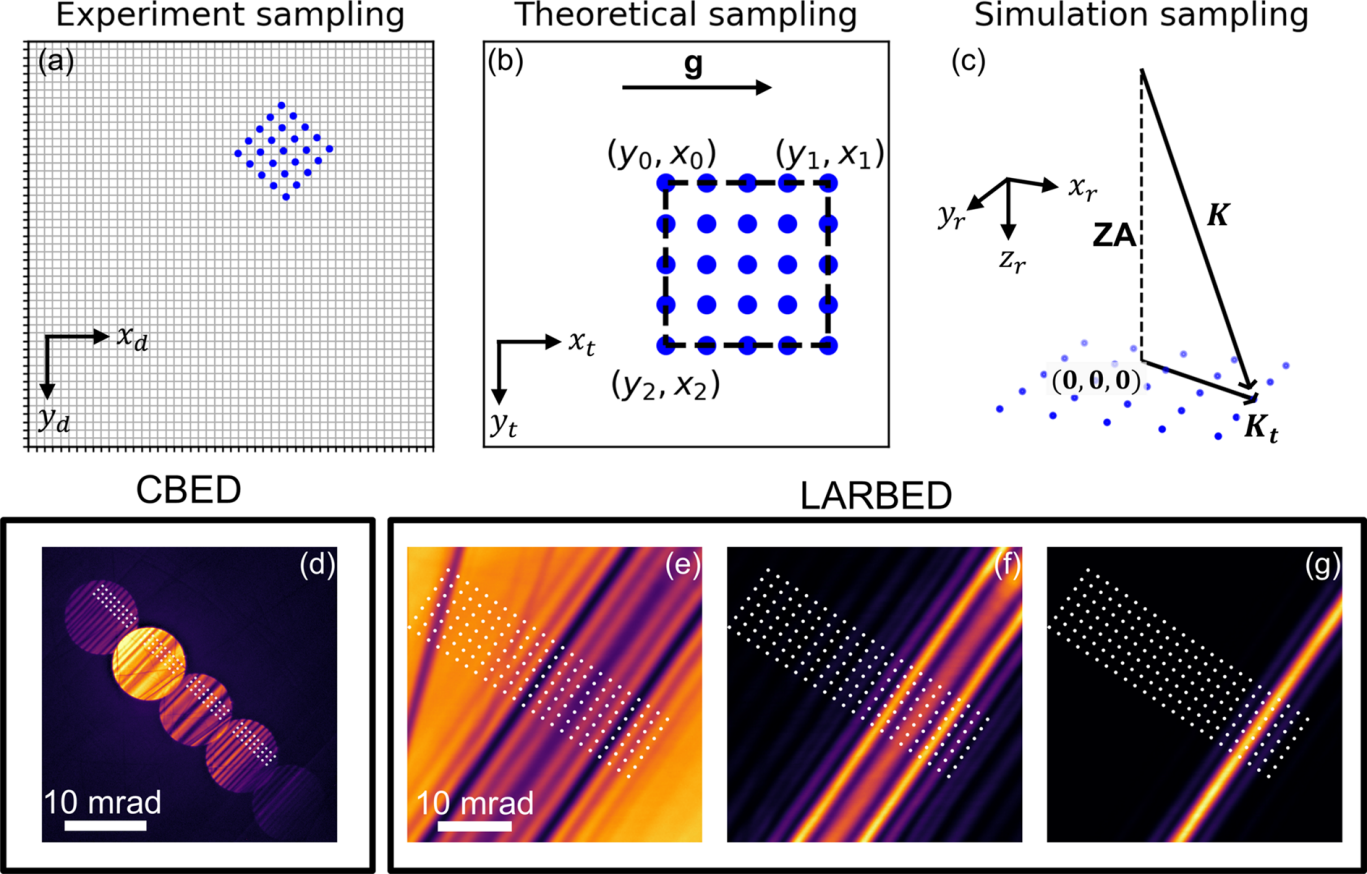
Illustration of three sampling schemes used in *PyExtal*. (*a*) Experiment sampling. Diffraction patterns are recorded and sampled in the pixel coordinates of the detector. (*b*) Simulation sampling. Diffraction patterns are rotated so a user-specified reciprocal lattice vector **g** is parallel to the horizontal axis. The user defines the region of interest in this coordinate system by specifying the three corners of a rectangle in detector pixel coordinates and the number of sampling points per side. (*c*) Sampling points in (*b*) are used to calculate the zone-axis coordinates. (*d*) An example of mapping simulation sampling onto the experimental sampling of CBED. (*e*) An example of experimental and simulation sampling for LARBED.

(1) *Experimental sampling coordinates* (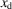, 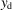). This is the raw data space. The sampling coordinates are defined by the pixel indices in the 2D image of the CBED or LACBED pattern, as illustrated in Fig. 2 ▸(*a*).

(2) *Theoretical sampling coordinates* (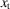, 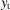). In this coordinate system, the experimental coordinates are rotated such that a reciprocal lattice vector (**g**) designated by the user is horizontal. The ROI for refinement is defined as a rectangle within this space. This is shown in Fig. 2 ▸(*b*).

(3) *Simulation sampling coordinates* (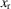, 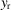). The Bloch wave simulations use the zone-axis coordinates defined by Spence & Zuo (1992 ▸) for the reciprocal space and the tangential wavevector 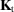 for the incident beam, as shown in Fig. 2 ▸(*c*).

*PyExtal* manages the transformations between these three coordinate systems. After the user defines an ROI and its sampling in the theoretical sampling coordinates, the coordinates are mapped into the experimental sampling coordinate system using the diffraction geometry parameters. Fig. 2 ▸(*d*) shows an example for CBED. Once the ROI is mapped, the experimental intensities at the sampling points are extracted for different reflections. Figs. 2 ▸(*e*)–2 ▸(*g*) illustrate how experimental intensities are extracted from the LACBED pattern of each reflection. Because the mapped coordinates on the experimental pattern are generally non-integers, spline interpolation functions are used to extract the intensity values. Similarly, the theoretical sampling coordinate system is transformed into zone-axis coordinates to calculate the theoretical intensities. This framework provides the flexibility to analyze both CBED and LACBED patterns within a unified workflow as the diffraction patterns are abstracted into arrays of intensities.

### 3.4. Coarse refinement: correlation-based optimization

The coarse refinement determines the parameters that primarily affect the positions of features in CBED patterns, such as high-order Laue zone (HOLZ) lines or rocking curve fringes. This is accomplished through a coarse refinement procedure that optimizes these parameters by maximizing the cross-correlation coefficient between the experimental and simulated patterns. In *PyExtal*, this is implemented as minimizing the output of a ‘target function’, which is the lowest correlation distance (*i.e.* 1 − correlation coefficient). To reduce the computational cost, simulation can be performed on a coarser sampling grid than the experimental sampling and subsequently up-sampled using interpolation.

Several optimizable target functions are pre-defined for the following parameters:

(1) *Geometric parameters*: crystal thickness, camera length and orientation. The crystal thickness or camera length is optimized in tandem with orientation. The orientation is refined by simulating a large-field-of-view pattern and using the ROI pattern as a template in searching for the position that maximizes the cross-correlation.

(2) *High voltage*: the microscope’s accelerating voltage, which directly influences the Ewald sphere curvature and thus the position of sensitive features like HOLZ lines.

(2) *Unit-cell parameters*: the six lattice parameters, which collectively determine the geometry of the entire diffraction pattern.

(4) *Debye–Waller factor* (*B*): an isotropic displacement parameter that accounts for atomic thermal vibrations, which primarily damps the intensity of high-order reflections and thus the transmitted beam intensity.

While the above discussion focuses on matching intensity features for refinement, there is also an option to filter the CBED patterns for feature-enhanced pattern matching (Zuo *et al.*, 1998 ▸) to improve the robustness of refinement. This can be done by providing a custom image preprocessing function that is applied to enhance specific features before the comparison between the experimental and simulated patterns is made. In the following subsection, we demonstrate an application example.

### 3.5. Thickness and orientation refinement

An accurate determination of the sample thickness and precise crystal orientation relative to the incident electron beam are prerequisites for further quantitative refinement. We demonstrate this capability using a LARBED dataset from a silicon crystal (space group 227, *a* = *b* = *c* = 5.4307 Å). The same method can be applied to CBED datasets.

**Figure 3:**
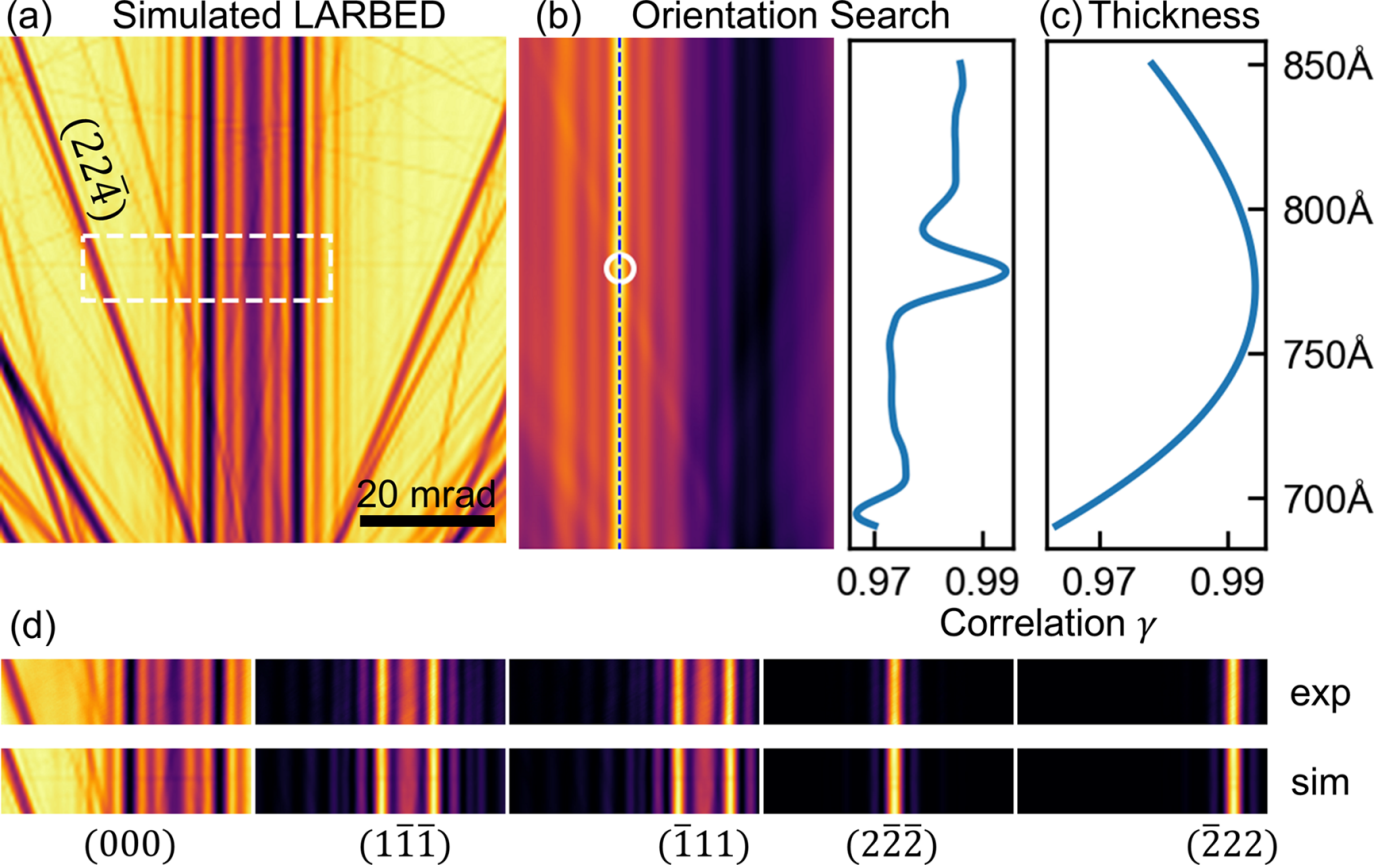
Coarse refinement with Si 111 systematic pattern. (*a*) Simulated LACBED pattern with field of view larger than the experiment for pattern matching. (*b*) Correlation map and profile along the dashed blue line. A clear peak can be identified in the profile for precise orientation search. (*c*) Correlation profile for different thickness. (*d*) Final matching results of all five reflections included in the refinement.

The workflow for this refinement is illustrated in Fig. 3 ▸. The process begins by selecting which reflections will be included in the refinement, in this case, 000, 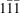, 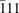, 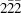 and 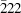. Subsequently, an ROI is selected, which is the same region as shown in Figs. 2 ▸(*e*)–2 ▸(*g*). The extracted intensities are shown in the first row of Fig. 3 ▸(*d*). The ROI is selected to contain distinct features, including inclined features, such as the prominent fringes of the rocking curve of 22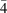 in Fig. 3 ▸(*a*), which serves as a marker to locate the ROI precisely.

Next, a Bloch wave simulation is performed as a reference of the sample orientation. This simulation covers a wider range of incident beam directions than is covered in the ROI, as the simulated LACBED pattern of the transmitted beam shows [Fig. 3 ▸(*a*)]. (Other reflections included in the fitting were also simulated but are not shown here.) The extracted experimental ROI patterns from the included reflections are then used as a 3D template to perform a cross-correlation search across the corresponding simulated patterns. The result of this search is a correlation map [Fig. 3 ▸(*b*)], where the value at each point corresponds to the correlation coefficient. The peak in this map, marked by a circle, identifies the orientation in the simulation that best matches the experiment, thereby determining the precise incident beam orientation of each pixel in the experimental pattern. A sharp peak is present in the line profile [Fig. 3 ▸(*b*)], showing that, with a distinctive feature like the 22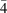 fringe, high precision in crystal orientation determination can be achieved.

The above template matching method is implemented as a target function taking the thickness as the variable and returning the correlation distance. The correlation map is generated with the function template_matching in the *scikit-image* library (Walt *et al.*, 2014 ▸). The optimization algorithm, in this case Brent’s method (Press *et al.*, 1988 ▸), varies the sample thickness, minimizing the output of the target function. The correlation coefficient profile is shown in Fig. 3 ▸(*d*). The profile is smooth because the features in LARBED patterns change gradually as the thickness increases.

The final result of this combined orientation and thickness optimization is validated by the good agreement between the experimental pattern and the simulation using the refined parameters. The bottom panels of Fig. 3 ▸ show a side-by-side comparison for all included reflections. The precise alignment of the rocking curve fringes across all reflections confirms the accuracy of the coarse refinement procedure. This rapid and robust optimization provides the starting point for subsequent, more computationally intensive, fine refinement of structure factors.

### 3.6. Structure factor refinement

Following the coarse refinement of the geometric and experimental parameters, a fine refinement stage is initiated to precisely determine structure factors. This stage directly compares the intensities of the experimental and simulated patterns on a pixel-by-pixel basis within the defined ROI. The optimization objective is to minimize a GOF metric.

The fine refinement treats the structure factors as parameters. To ensure numerical stability, the structure factors are normalized to a user-defined range during optimization. The refinement is iterative: in each main iteration, the structure factors are adjusted, and equation (5) is solved, returning the corresponding eigenvalues and eigenvectors. Subsequently, an inner optimization loop refines the sample thickness in the intensity calculation [equation (4)] and geometric parameters for ROI mapping to achieve the best possible fit for the newly proposed set of structure factors. Among the geometric parameters, offsets are included, allowing ROIs to displace separately for each reflection. This is used to compensate for small geometric distortions in the CBED patterns, induced by aberrations in the post-specimen lenses. The optimization process is driven by the Nelder–Mead method implemented in *scipy* (Virtanen *et al.*, 2020 ▸).

The default GOF is the 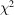 metric, as defined in equation (1). Several variations of the 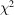 metric are implemented to accommodate different experimental conditions:

(1) chi2_const: assumes a constant background and uses detector characteristics to model the variance.

(2) chi2_multibackground: allows for different background levels for different diffraction discs, which is useful for CBED patterns with detector artifacts.

(3) chi2_LARBED: uses a pre-calculated pixel-wise variance map for LARBED data, where noise estimation can be more complex.

(4) Custom GOF. Users can define their own goodness-of-fit metrics by inheriting from the BaseGOF class and implementing a custom scoring method. This provides extensibility for specialized refinement problems.

For LARBED data, the model can be further improved by convolving the simulated pattern with a 2D Voigt function to model the beam profile in reciprocal space resulting from the finite convergence angle of the probe. The parameters of this Voigt function are refined along with the geometric parameters in the inner optimization loop.

## 4. Comparison with *Extal*: using Si structure factor refinement as an example

### 4.1. Structure factor refinement of Si 111 systematic reflections with CBED

**Figure 4:**
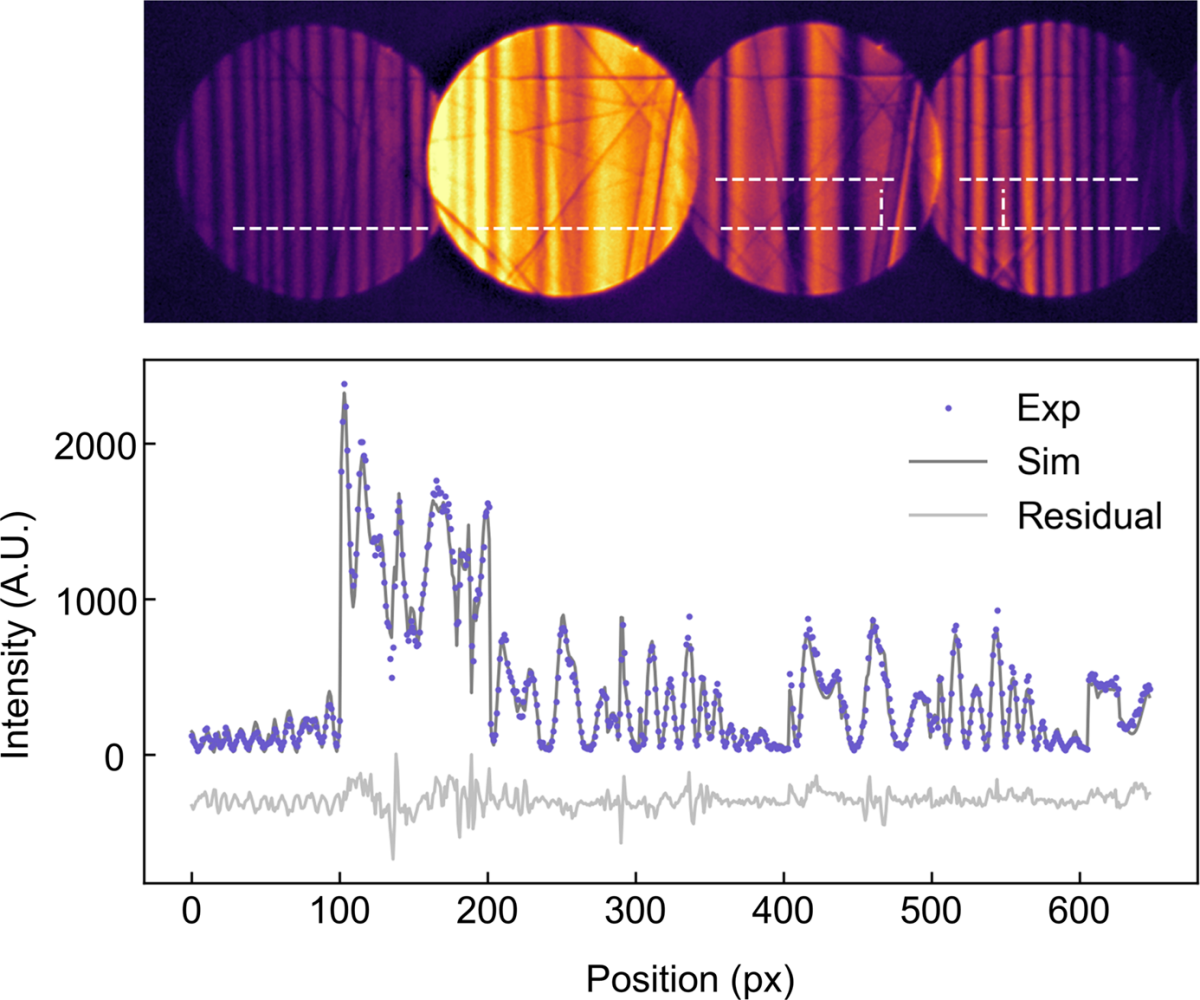
(*a*) Si 111 systematic CBED pattern. The pattern is energy-filtered and deconvoluted with detector MTF. The intensity is square rooted to show the details in dark-field discs. (*b*) Comparison of experimental intensities along the white lines shown in (*a*) with simulated intensities calculated with refined structure factor, and the residual difference.

We used an energy-filtered Si 111 systematic row CBED pattern to demonstrate the ability of *PyExtal* to refine CBED patterns and compare the result with that from *Extal*. This dataset was previously published by Zuo (1998 ▸). Before refinement of structure factors, a coarse refinement as described in Section 3.4 was performed to establish the orientation of the diffraction pattern. The same ROIs were used for both *Extal* and *PyExtal* refinements for comparison. The selected ROI is shown in Fig. 4 ▸(*a*). The experimental and simulated intensities, along with their difference, are plotted in Fig. 4 ▸(*b*). The fit result is similar to Fig. 8 of Zuo (1998 ▸). The fitted structure factors are shown in Table 1 ▸. The good agreement demonstrates the reliability of the refinement results obtained using *PyExtal*.

### 4.2. Structure factor refinement of Si 111 systematic reflections with LARBED

**Figure 5:**
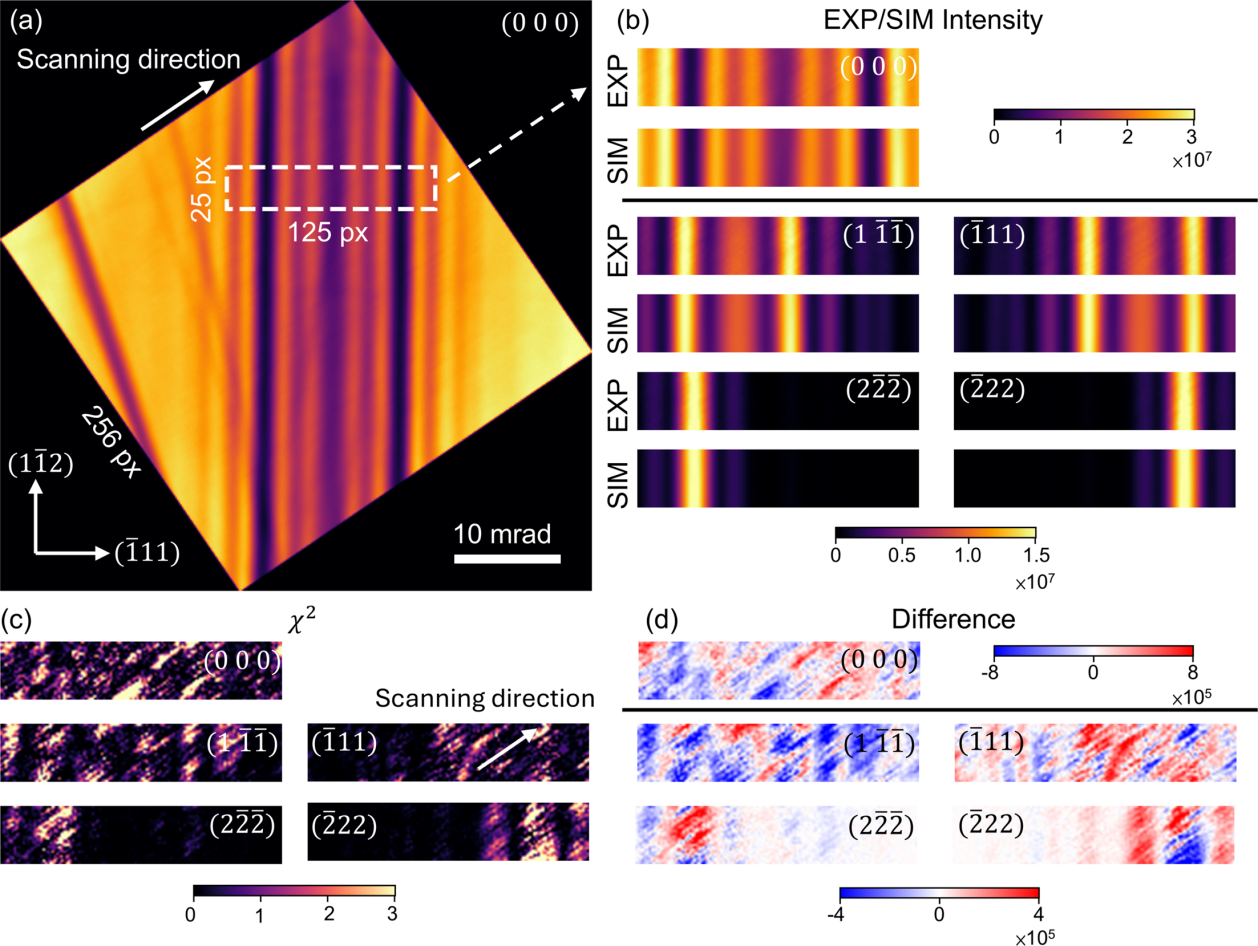
Results of structure factor refinement of Si 111 systematic pattern. (*a*) The LACBED pattern of 000. The 125 × 25 pixel region in the white box is selected for refinement. (*b*) Experimental intensities extracted from the ROI and the intensities simulated with the refined structure factors. (*c*) 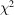 map for each incident beam direction. Scanning artifacts can be clearly seen along the scanning direction. (*d*) Difference map for each incident beam direction.

Here we demonstrate that the same level of accuracy in structure factor refinement as obtained with CBED can be achieved with LARBED. We proceed to refine the structure factors (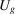) for the Si 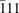 and 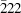 reflections with the off-zone-axis Si LACBED patterns shown in Section 3.5. The refined region is the area enclosed by the thick dashed white line in Fig. 5 ▸(*a*). The theoretical intensities were convolved with a 2D Voigt function to account for the small convergence of the incident beam in LARBED. The function parameters were optimized concurrently with in-plane rotation, camera length, orientation and thickness.

The refinement was initialized using the IAM values for the structure factor, with a modification for the forbidden reflection: the starting value for 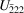 was set to 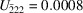
 Å^−2^ with a phase of 180°, while 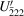 was kept as zero throughout the refinement. The optimization converged to a final 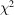 value of 0.556. A value less than unity suggests that the variance of the experimental data was probably overestimated.

Structure factors obtained with the IAM model, X-ray diffraction, CBED and LARBED are compared in Table 2 ▸. The deviation of the refined 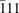 and 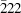 structure factor values from the IAM value is attributed to crystal bonding effects. The final non-zero value for the forbidden 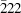 reflection in IAM provides a direct measurement of the charge density redistribution due to covalent bonding in silicon. The value of 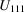 measured by LARBED is in excellent agreement with the X-ray and CBED results, and a slightly different value of 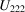 is obtained with LARBED.

The quality of the fit is illustrated in Fig. 5 ▸(*b*). The best-fit simulations and their residuals demonstrate that the model accurately reproduces the features of the rocking curves across all analyzed reflections. This confirms the effectiveness of the fine refinement procedure in *PyExtal* for extracting accurate structure factor information.

## 5. Application to large-unit-cell crystal: structure factor refinement of yttrium iron garnet

A critical advantage of the LARBED method is the simultaneous collection of LACBED patterns for many reflections, which enables the study of large-unit-cell crystals. To show the potential of LARBED for structure factor refinement of large-unit-cell crystals, we use yttrium iron garnet (YIG, space group 230, 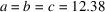
 Å) as a demonstration example.

**Figure 6:**
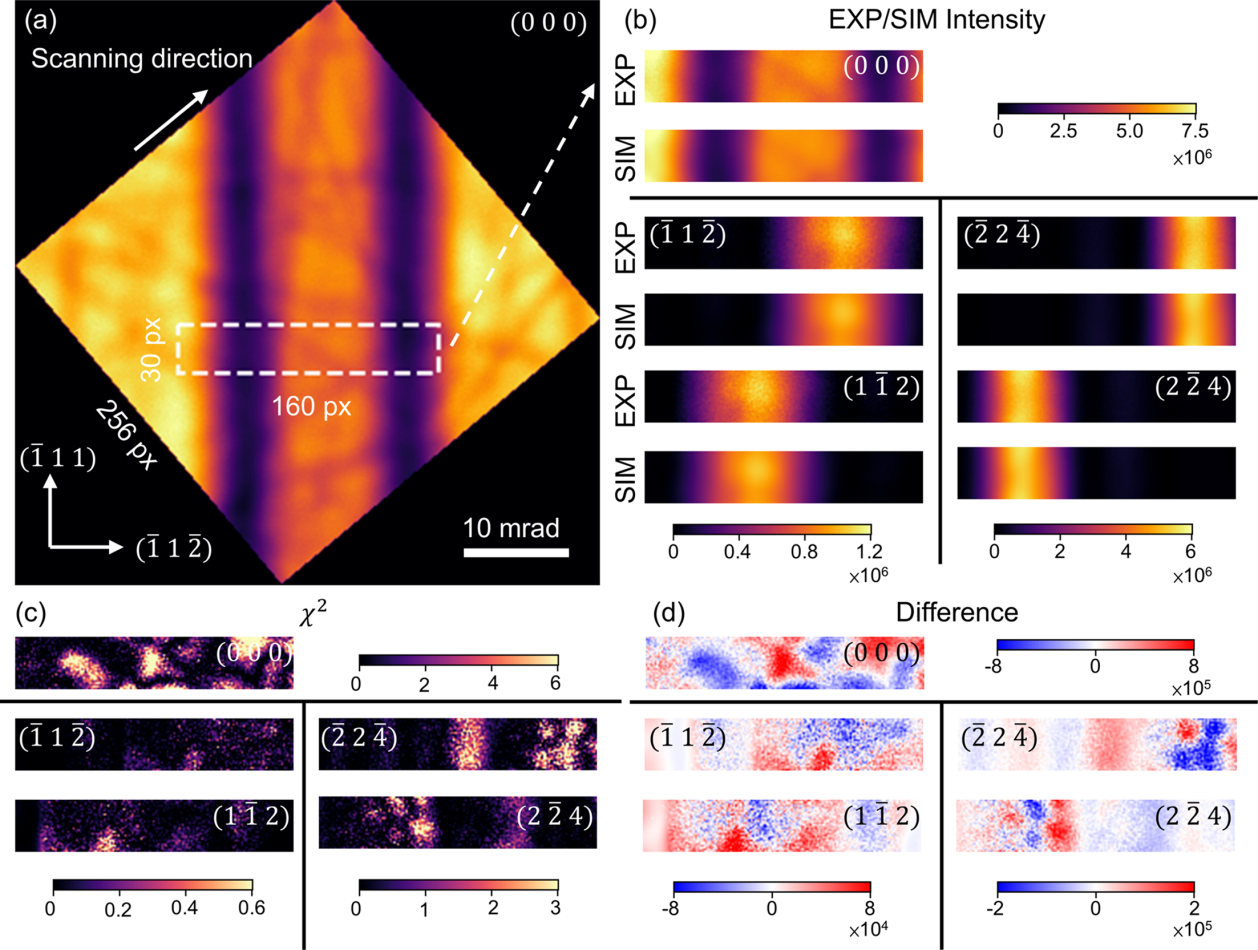
Results of structure factor refinement of YIG 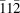 systematic pattern. (*a*) YIG LACBED pattern of 000. The 160 × 30 pixel region in the white box is selected for refinement. (*b*) Experimental intensities extracted from the ROI and the intensities simulated with the refined structure factors. (*c*) 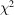 map for each incident beam direction. Shot noise can be seen in the map. (*d*) Difference map for each incident beam direction.

Fig. 6 ▸ shows the LACBED pattern of the transmitted beam in a LARBED dataset collected from a YIG crystal sample, off the [110] zone axis with a near-systematic diffraction condition. The experiment was performed at an electron acceleration voltage of 300 kV using the Themis Z scanning/transmission electron microscope installed at the University of Illinois. The microscope is installed inside a custom-designed mu-metal box with excellent environmental isolation. The 000, 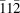, 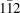, 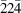 and 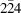 reflections are included in the dataset. Following the practice established in Section 3.5, the orientation of the systematic off-zone-axis orientation was determined first using the selected ROI for coarse refinement, as shown in Fig. 6 ▸(*a*). The ROI is chosen to include features with multiple intensity deficiencies in the 000 disc, covering the rocking curve fringes of the 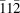 and 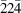 reflections. These features, particularly in the 000 disc, allow accurate determination of the tilt along the systematic excitation. Template matching, as discussed in Section 3.5, identifies the incident beam orientation and thickness, resulting in a near match of the initial Bloch wave simulation [Fig. 6 ▸(*b*)].

After determining the incident beam orientation and thickness, we refined the structure factors (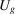) for the YIG 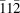 and 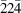 reflections. The starting structure factor values for all reflections are initialized using the IAM values. After refinement, the optimization converged to a final 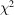 value of 0.5563. The refined structure factors for the two systematic reflections resolve to


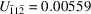
 Å^−2^, 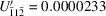
 Å^−2^ and


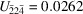
 Å^−2^, 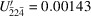
 Å^−2^.

The final fitting results are shown in Fig. 6 ▸(*b*). The highly accurate fitting of rocking curves is reflected in the intensity match in both reflections and marks a promising step forward for QCBED of large-unit-cell crystals.

## 6. Discussion

We have implemented *PyExtal* as a Python-based refinement solution for QCBED and demonstrated that accurate structure factors can be measured from both CBED and LARBED datasets using *PyExtal*. The ability to refine LARBED datasets has the distinct advantage of being applicable to large-unit-cell crystals and beam-sensitive materials.

### 6.1. Computational requirements for refinement

**Figure 7:**
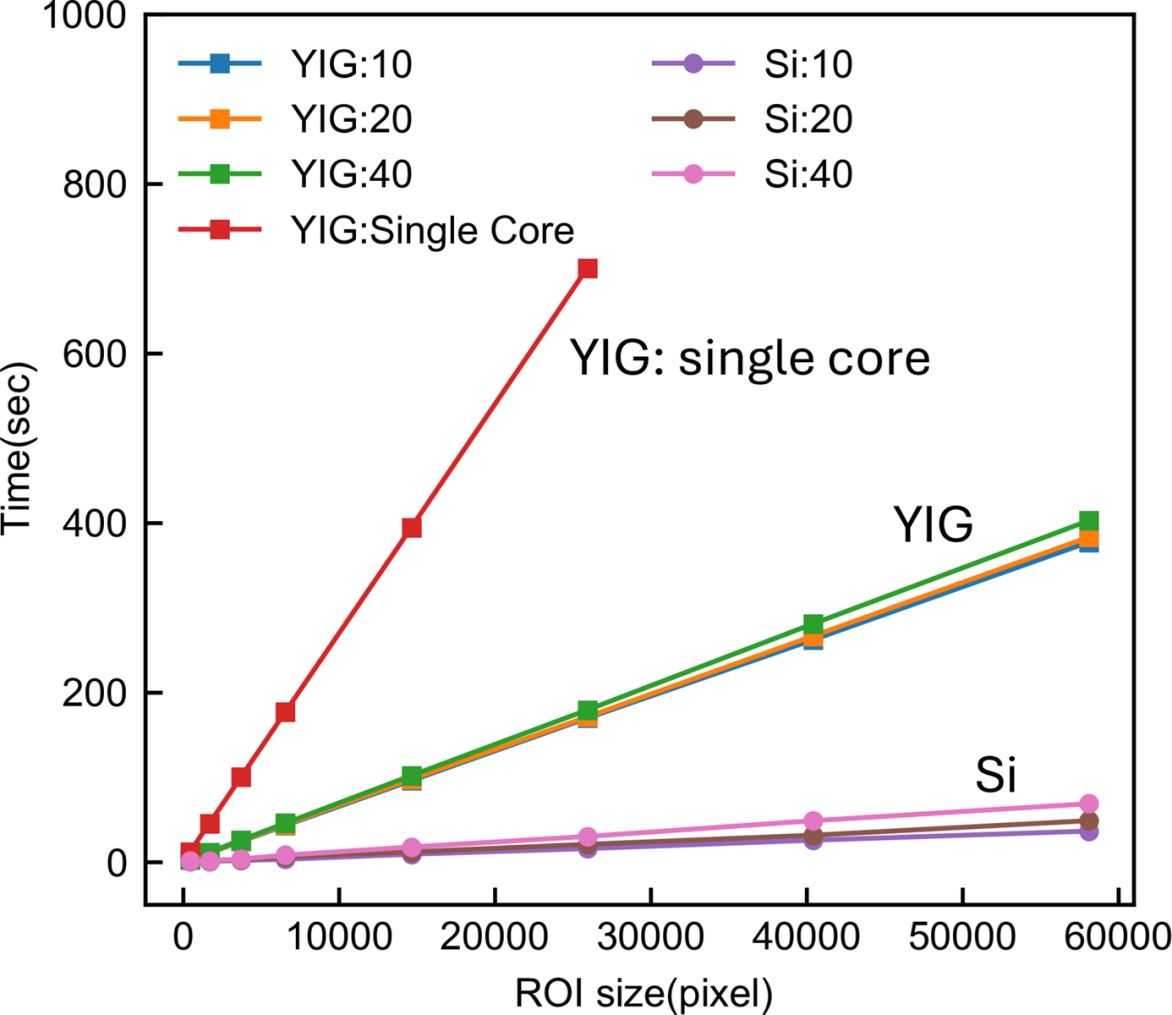
Benchmark for different simulation parameters. The average diagonalization matrix size for YIG is 166 × 166 and for Si is 70 × 70.

The computational performance of *PyExtal* is largely determined by the computational resources available for the Bloch wave simulation. We benchmarked the performance of Bloch wave simulation using silicon and YIG as examples on a personal computer equipped with an Intel i7-12700 CPU, with *PyExtal* running on the Windows Subsystem for Linux (WSL). All simulations utilized eight parallel processes except one in which we used a single core for the YIG example (YIG: single core profile). The results, presented in Fig. 7 ▸, demonstrate the impact of simulation parameters and parallel processing on computation time. For the Si example, a total of 363 beams were included in the Bloch wave simulation, resulting in an average diagonalization matrix size of approximately 70 × 70 after applying the perturbation treatment of the Bethe potential. For the YIG example, 676 beams were included and the average diagonalization matrix size was 166 × 166. With the default stopping criteria, including 493 beams in the calculation and sampling 6561 incident beam directions, it takes 44 s to perform the thickness and orientation refinement on the CBED example shown in Fig. 4 ▸. For the structure factor, 244 incident beam directions were used, and it took 183 s to refine. The stopping criteria and number of beams included can be easily adjusted by the user for trade-off between computation time and precision.

The computation time, defined as the duration for one complete matrix diagonalization and intensity calculation cycle, is influenced by three main factors. The time scales linearly with both the number of pixels in the region of interest and the number of output beams for which intensities are calculated. However, the most significant factor is the number of beams (**g**) included in equation (5) (*N*), as the diagonalization step has a time complexity of 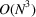. This cubic scaling explains the substantially longer computation times required for YIG compared with silicon. To accelerate the simulation, the eigen-problem tasks are parallelized. By considering the profiles of the single core and eight cores, we measured a 4 times speed improvement after parallelization. The parallelization architecture of *PyExtal* is built on the MPI, allowing it to be scaled up for use on high-performance computing clusters and supercomputers. Access to such resources in the future could alleviate the raw computational limits.

The speed of the Bloch wave simulation cycle is critical, as a typical refinement requires hundreds of matrix diagonalizations and tens of thousands of intensity calculations. Therefore, optimizing the experimental geometry for computational efficiency can be highly advantageous. We find off-axis orientations are generally preferable to zone-axis orientations for several reasons. First, zone-axis patterns exhibit strong multi-beam excitation, which increases the number of structure factors that must be refined simultaneously. This expands the dimensionality of the optimization space, leading to a significant increase in total refinement time. Secondly, at zone-axis conditions, more beams are excited, requiring a larger diagonalization matrix to accurately simulate the diffraction intensity. The large number of structure factors that can be refined from zone-axis patterns could be advantageous as it simplifies diffraction data collection. However, realization of this advantage requires the development of robust optimization methods for a large set of parameters.

For crystals with very large unit cells and dense reciprocal lattices, the fundamental complexity of multi-beam refinement remains a challenge. Future work could address this by exploring new strategies for dynamical diffraction simulation, including the use of the multi-slice method of Cowley & Moodie (1957 ▸).

### 6.2. Quality of LARBED datasets

While energy-filtered CBED is a well established experimental method (Midgley *et al.*, 1995 ▸; Holmestad *et al.*, 1999 ▸; Mayer *et al.*, 1995 ▸), the experimental setup for LARBED is still under development. To elevate LARBED into a routine quantitative technique, several hardware and methodological advancements will be crucial. First, as LARBED datasets are acquired serially, the quality is inherently more susceptible to microscope instabilities, so improving the instrument stability is critical. For instance, the Si dataset presented in Section 4.2 exhibits stripe-like features along the scanning direction, as shown in Fig. 5 ▸. This artifact is likely to be the result of incident beam movement during rocking, leading to minor inconsistencies in sample thickness and orientation.

To further investigate the LARBED data quality issue, we acquired four independent LARBED datasets from the same Si TEM sample to investigate the consistency of the structure factor refinement. For each dataset, five different ROIs, each 125 × 5 pixels, were selected for refinement. These ROIs cover the same horizontal range as shown in Fig. 5 ▸ but are sampled from different vertical positions. The refinement was performed using parameters identical to those described in Section 4.2.

The results for all 20 refinements are summarized in Table 3 ▸. The refined thickness shows excellent consistency, with a standard deviation of less than 0.6% of the mean value for each dataset. A similarly low variation is observed for the strong 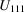 structure factor, where the standard deviation within a single dataset is less than 0.3%. The standard deviation comparing the four datasets is slightly higher, at approximately 0.4%. In contrast, the weaker structure factors exhibit higher relative standard deviation. This is expected for the weak 222 reflection, as its intensity is an order of magnitude smaller than that of the primary reflections. Similarly, the absorptive structure factor 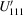, which accounts for the absorption effect, is much weaker than its elastic counterpart and thus more susceptible to noise. Despite these variations in the weaker parameters, the low 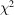 values for all refinements indicate that the model is consistently a good fit to the data. Overall, this analysis demonstrates that *PyExtal* produces highly reliable and reproducible results across different LARBED datasets and ROIs.

### 6.3. Future prospects of LARBED for quantitative electron diffraction

Looking forward, LARBED could be integrated with 3DED. Structure refinements from 3DED often yield higher *R* factors relative to X-ray methods due to dynamical diffraction effects (Klar *et al.*, 2023 ▸). Integrating precession electron diffraction and dynamic diffraction calculation into the structure refinement workflow improves the refinement *R* factor and the precision of structural parameters (Palatinus *et al.*, 2015*a* ▸; Palatinus *et al.*, 2015*b* ▸; Klar *et al.*, 2023 ▸; Plana-Ruiz *et al.*, 2025 ▸). In comparison, CBED or LARBED measures rocking curves at high resolution (0.2 mrad or smaller). Using this additional information, one can quantify the thickness and crystal orientation very precisely. By fully accounting for dynamic scattering within the refinement, this approach promises more accurate structures and significantly lower *R* factors (Cleverley & Beanland, 2023 ▸), providing high-quality structure factors for resolving the charge-density distribution. The LARBED–3DED combination offers a powerful solution that can leverage the advantage of reliable fitting with the structure determination of 3DED. A dedicated microscope designed for such stage-tilting experiments would also streamline data collection across multiple zone axes, enabling more efficient acquisition of the comprehensive datasets required for multipole refinement or Fourier synthesis of electron density maps.

Another consideration is the use of an energy filter. While the LARBED background subtraction strategy used in this work yields results comparable to results obtained with energy-filtered CBED, a quantitative comparison with energy-filtered data is still needed. In our dataset, we observed that thicker samples can exhibit anomalous intensity asymmetries in the LARBED patterns. An energy filter removes the strong inelastic background in thick samples, so we expect an improvement in LARBED data quality.

Detector technology also plays a pivotal role. The development of direct electron detectors has already enabled detection with a large dynamic range and high detector quantum efficiency. The EMPAD used in this work (Tate *et al.*, 2016 ▸) has a 128 × 128 pixel format and a 1000 fps frame rate. Future detectors with larger formats would allow more diffraction spots to be captured simultaneously, offering greater flexibility in the selection of camera length and providing more data points for background fitting. Higher frame rates would accelerate data acquisition, reducing the impact of sample drift and other sample-related issues.

We have modernized the structure factor refinement algorithm with a user-friendly Python interface and parallel computing. Future developments include GPU-accelerated computation, direct refinement of structure models and adaptation of new optimization algorithms, especially those developed for deep learning (Kingma & Ba, 2017 ▸). Features such as support for .cif file input or Conda packages for multiple OS platforms are also under consideration. We welcome feedback and contributions from the diffraction community.

## 7. Conclusion

We have developed a Python package for quantitative CBED called *PyExtal*. This package couples the programming flexibility of Python scripting with an efficient Fortran-based Bloch wave simulation engine for structural parameter refinement. For CBED, this hybrid computational approach yields both accuracy and flexibility, facilitating robust workflows for quantitative electron diffraction analysis. For LARBED, *PyExtal* enables the first demonstration of quantitative refinement in large-unit-cell crystals, which was not possible using CBED. This approach opens a whole new possibility of using electron diffraction to investigate the valance electrons and bonding inside large-unit-cell crystals with high accuracy.

## Figures and Tables

**Table 1:** Refined structure factor for Si 111 and 222 at 200 kV using CBED

Method	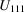	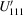	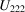	GOF
*Extal*	0.04731	0.00086	0.00093	4.2
*PyExtal*	0.04735	0.00079	0.00092	2.8

**Table 2:** Refined structure factor for Si 111 and 222 at 300 kV

Method	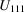	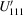	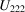	GOF
IAM	0.05749	0.00047	0	–
X-ray (Zuo *et al.*, 1997 ▸)	0.05434	–	0.001082	–
CBED	0.05430	0.00091	0.001055	2.8
LARBED	0.05449	0.00067	0.001191	0.6

**Table 3:** Structure factor of Si 111 and 222 from different regions

	Thickness (Å)	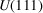	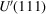	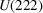	GOF
Dataset 1	805 (2)	0.05444 (10)	0.00072 (3)	0.00119 (19)	0.527 (83)
Dataset 2	661 (2)	0.05395 (17)	0.00090 (3)	0.00115 (10)	0.870 (99)
Dataset 3	726 (4)	0.05422 (14)	0.00078 (7)	0.00120 (10)	1.127 (284)
Dataset 4	661 (1)	0.05409 (7)	0.00094 (4)	0.00120 (6)	0.645 (125)
Average	–	0.05418 (22)	0.00084 (10)	0.00118 (11)	–

## Data Availability

The source code and Si data are available at https://github.com/swampni/PyExtal and via the Zenodo page of *PyExtal* (Ni *et al.*, 2025 ▸). The Python portion of the code is licensed under MPL 2.0.

## References

[bb50] Anderson, E., Bai, Z., Bischof, C., Blackford, S., Demmel, J., Dongarra, J., Du Croz, J., Greenbaum, A., Hammarling, S., McKenney, A. & Sorensen, D. (1999). *LAPACK Users’ Guide*, 3rd ed. Society for Industrial and Applied Mathematics.

[bb1] Beanland, R., Thomas, P. J., Woodward, D. I., Thomas, P. A. & Roemer, R. A. (2013). *Acta Cryst.* A**69**, 427–434. 10.1107/S0108767313010143 PMC3686228 23778099

[bb2] Birkeland, C., Holmestad, R., Marthinsen, K. & Høier, R. (1998). *J. Sci. Comput.* **13**, 1–18.

[bb3] Busch, R., Ni, H.-C., Shao, Y.-T. & Zuo, J.-M. (2024). *Microsc. Microanal.* **31**, ozae088. 10.1093/mam/ozae088 39353861

[bb4] Cleverley, A. & Beanland, R. (2023). *IUCrJ* **10**, 118–130. 10.1107/S2052252522011290 PMC9812222 36598507

[bb5] Cowley, J. M. & Moodie, A. F. (1957). *Acta Cryst.* **10**, 609–619.

[bb7] Dominiak, P., Kumar, A., Suresh, A., Lanza, A., Wojciechowski, J., Trzybiński, D., Brazda, P. & Palatinus, L. (2025). *Experimental Charge Density of Organic Nanocrystals Revealed by 3D Electron Diffraction*, https://www.researchsquare.com/article/rs-7433721/v1.

[bb8] Doyle, P. A. & Turner, P. S. (1968). *Acta Cryst.* A**24**, 390–397.

[bb9] Friis, J., Jiang, B., Spence, J. C. & Holmestad, R. (2003). *Microsc. Microanal.* **9**, 379–389. 10.1017/s1431927603030319 19771694

[bb10] Gemmi, M., Mugnaioli, E., Gorelik, T. E., Kolb, U., Palatinus, L., Boullay, P., Hovmöller, S. & Abrahams, J. P. (2019). *ACS Cent. Sci.* **5**, 1315–1329. 10.1021/acscentsci.9b00394 PMC6716134 31482114

[bb11] Holmestad, R., Birkeland, C. R., Marthinsen, K., Høier, R. & Zuo, J. M. (1999). *Microsc. Res. Tech.* **46**, 130–145. 10.1002/(SICI)1097-0029(19990715)46:2<130::AID-JEMT6>3.0.CO;2-O 10423558

[bb12] Jiang, B., Zuo, J.-M., Friis, J. & Spence, J. C. (2003). *Microsc. Microanal.* **9**, 457–467. 10.1017/s1431927603030381 19771701

[bb13] Jones, C. G., Martynowycz, M. W., Hattne, J., Fulton, T. J., Stoltz, B. M., Rodriguez, J. A., Nelson, H. M. & Gonen, T. (2018). *ACS Cent. Sci.* **4**, 1587–1592. 10.1021/acscentsci.8b00760 PMC6276044 30555912

[bb14] Kingma, D. P. & Ba, J. (2017). *arXiv*,1412.6980 [cs]. https://arxiv.org/abs/1412.6980.

[bb15] Klar, P. B., Krysiak, Y., Xu, H., Steciuk, G., Cho, J., Zou, X. & Palatinus, L. (2023). *Nat. Chem.* **15**, 848–855. 10.1038/s41557-023-01186-1 PMC10239730 37081207

[bb16] Koch, C. T. (2011). *Ultramicroscopy* **111**, 828–840. 10.1016/j.ultramic.2010.12.014 21227590

[bb17] Kolb, U., Gorelik, T., Kübel, C., Otten, M. T. & Hubert, D. (2007). *Ultramicroscopy* **107**, 507–513. 10.1016/j.ultramic.2006.10.007 17234347

[bb18] Lin, W., Xue, Z., Cui, W., Kulovits, A., Ren, H., Zhao, W., Wu, J., Van Tendeloo, G., Wiezorek, J. & Sang, X. (2025). *Nat. Commun.* **16**, 5811. 10.1038/s41467-025-60966-0 PMC12214857 40592890

[bb19] Mahmoudi, S., Gruene, T., Schröder, C., Ferjaoui, K. D., Fröjdh, E., Mozzanica, A., Takaba, K., Volkov, A., Maisriml, J., Paunović, V., van Bokhoven, J. A. & Keppler, B. K. (2025). *Nature* **645**, 88–94. 10.1038/s41586-025-09405-0 PMC12408337 40836092

[bb6] Mayer, J., Deininger, C. & Reimer, L. (1995). *Electron Spectroscopic Diffraction*, in *Energy-Filtering Transmission Electron Microscopy*, Springer Series in Optical Sciences, Vol. 71, edited by L. Reimer. Springer.

[bb20] Midgley, P. A., Saunders, M., Vincent, R. & Steeds, J. W. (1995). *Ultramicroscopy* **59**, 1–13.

[bb21] Nakashima, P. N. H. & Muddle, B. C. (2010). *Phys. Rev. B* **81**, 115135.

[bb22] Nakashima, P. N. H., Smith, A. E., Etheridge, J. & Muddle, B. C. (2011). *Science* **331**, 1583–1586. 10.1126/science.1198543 21436448

[bb23] Ni, H.-C., Busch, R. & Zuo, J.-M. (2025). s*wampni/PyExtal: Python Package for Quantitative Electron Crystallography*, https://zenodo.org/records/17836589. 10.1107/S1600576726001469 PMC13060613 41959864

[bb24] Nüchter, W., Weickenmeier, A. L. & Mayer, J. (1998). *Acta Cryst.* A**54**, 147–157.

[bb25] Ogata, Y., Tsuda, K., Akishige, Y. & Tanaka, M. (2004). *Acta Cryst.* A**60**, 525–531. 10.1107/S0108767304016307 15507734

[bb26] Palatinus, L., Corrêa, C. A., Steciuk, G., Jacob, D., Roussel, P., Boullay, P., Klementová, M., Gemmi, M., Kopeček, J., Domeneghetti, M. C., Cámara, F. & Petříček, V. (2015*a*). *Acta Cryst.* B**71**, 740–751. 10.1107/S2052520615017023 26634732

[bb27] Palatinus, L., Petříček, V. & Corrêa, C. A. (2015*b*). *Acta Cryst.* A**71**, 235–244. 10.1107/S2053273315001266 25727873

[bb28] Peterson, P. (2009). *Int. J. Comput. Sci. Eng.* **4**, 296–305.

[bb29] Plana-Ruiz, S., Lu, P., Ummethala, G. & Dunin-Borkowski, R. E. (2025). *J. Appl. Cryst.* **58**, 1249–1260. 10.1107/S1600576725005606 PMC12321033 40765972

[bb30] Press, W. H., Flannery, B. P., Teukolsky, S. A. & Vetterling, W. T. (1988). *Numerical Recipes in C.* Cambridge University Press.

[bb31] Saunders, M., Bird, D. M., Zaluzec, N. J., Burgess, W. G., Preston, A. R. & Humphreys, C. J. (1995). *Ultramicroscopy* **60**, 311–323.

[bb32] Saunders, M., Fox, A. G. & Midgley, P. A. (1999). *Acta Cryst.* A**55**, 480–488. 10.1107/s0108767398016316 10926691

[bb33] Spence, J. C. H. & Zuo, J. M. (1992). *Electron Microdiffraction.* Boston: Springer US.

[bb34] Tate, M. W., Purohit, P., Chamberlain, D., Nguyen, K. X., Hovden, R., Chang, C. S., Deb, P., Turgut, E., Heron, J. T., Schlom, D. G., Ralph, D. C., Fuchs, G. D., Shanks, K. S., Philipp, H. T., Muller, D. A. & Gruner, S. M. (2016). *Microsc. Microanal.* **22**, 237–249. 10.1017/S1431927615015664 26750260

[bb35] Tsuda, K. & Tanaka, M. (1999). *Acta Cryst.* A**55**, 939–954. 10.1107/s0108767399005401 10927304

[bb37] van der Walt, S., Schönberger, J. L., Nunez-Iglesias, J., Boulogne, F., Warner, J. D., Yager, N., Gouillart, E., Yu, T. & the *scikit-image* contributors (2014). *PeerJ* **2**, e453, https://peerj.com/articles/453. 10.7717/peerj.453 PMC4081273 25024921

[bb36] Virtanen, P., Gommers, R., Oliphant, T. E., Haberland, M., Reddy, T., Cournapeau, D., Burovski, E., Peterson, P., Weckesser, W., Bright, J., van der Walt, S. J., Brett, M., Wilson, J., Millman, K. J., Mayorov, N., Nelson, A. R. J., Jones, E., Kern, R., Larson, E., Carey, C. J., Polat, I., Feng, Y., Moore, E. W., VanderPlas, J., Laxalde, D., Perktold, J., Cimrman, R., Henriksen, I., Quintero, E. A., Harris, C. R., Archibald, A. M., Ribeiro, A. H., Pedregosa, F., van Mulbregt, P., Vijaykumar, A., Bardelli, A. P., Rothberg, A., Hilboll, A., Kloeckner, A., Scopatz, A., Lee, A., Rokem, A., Woods, C. N., Fulton, C., Masson, C., Häggström, C., Fitzgerald, C., Nicholson, D. A., Hagen, D. R., Pasechnik, D. V., Olivetti, E., Martin, E., Wieser, E., Silva, F., Lenders, F., Wilhelm, F., Young, G., Price, G. A., Ingold, G., Allen, G. E., Lee, G. R., Audren, H., Probst, I., Dietrich, J. P., Silterra, J., Webber, J. T., Slavič, J., Nothman, J., Buchner, J., Kulick, J., Schönberger, J. L., de Miranda Cardoso, J. V., Reimer, J., Harrington, J., Rodríguez, J. L. C., Nunez-Iglesias, J., Kuczynski, J., Tritz, K., Thoma, M., Newville, M., Kümmerer, M., Bolingbroke, M., Tartre, M., Pak, M., Smith, N. J., Nowaczyk, N., Shebanov, N., Pavlyk, O., Brodtkorb, P. A., Lee, P., McGibbon, R. T., Feldbauer, R., Lewis, S., Tygier, S., Sievert, S., Vigna, S., Peterson, S., More, S., Pudlik, T., Oshima, T., Pingel, T. J., Robitaille, T. P., Spura, T., Jones, T. R., Cera, T., Leslie, T., Zito, T., Krauss, T., Upadhyay, U., Halchenko, Y. O. & Vázquez-Baeza, Y. (2020). *Nat. Methods* **17**, 261–272.

[bb38] Wu, L., Meng, Q. & Zhu, Y. (2020). *Ultramicroscopy* **219**, 113095. 10.1016/j.ultramic.2020.113095 32905856

[bb39] Zhang, D., Oleynikov, P., Hovmöller, S. & Zou, X. (2010). *Z. Kristallogr.* **225**, 94–102.

[bb40] Zuo, J. (1999). *Microsc. Res. Tech.* **46**, 220–233. 10.1002/(SICI)1097-0029(19990801)46:3<220::AID-JEMT5>3.0.CO;2-1 10420176

[bb41] Zuo, J. M. (1998). *Mater. Trans. JIM* **39**, 938–946.

[bb42] Zuo, J. M. (2000). *Microsc. Res. Tech.* **49**, 245–268. 10.1002/(SICI)1097-0029(20000501)49:3<245::AID-JEMT4>3.0.CO;2-O 10816266

[bb43] Zuo, J. M., Blaha, P. & Schwarz, K. (1997). *J. Phys. Condens. Matter* **9**, 7541–7561.

[bb44] Zuo, J. M., Kim, M. & Holmestad, R. (1998). *J. Electron Microsc.* **47**, 121–127.

[bb45] Zuo, J. M., Kim, M., O’Keeffe, M. & Spence, J. C. H. (1999). *Nature* **401**, 49–52.

[bb46] Zuo, J. M. & Spence, J. C. H. (1991). *Ultramicroscopy* **35**, 185–196.

[bb47] Zuo, J. M. & Weickenmeier, A. L. (1995). *Ultramicroscopy* **57**, 375–383.

